# The role of parental involvement in fundamental movement skills among Hungarian school-aged children

**DOI:** 10.1186/s13102-025-01289-x

**Published:** 2025-08-14

**Authors:** Márton István Pelyvás, Klára Kovács

**Affiliations:** 1 MTA-DE-Parent-Teacher Cooperation Research Group, Institute of Sports Science, University of Debrecen, Egyetem sqr. 1, Debrecen, 4032 Hungary; 2 MTA-DE-Parent-Teacher Cooperation Research Group, Institute of Educational Studies and Cultural Management, Faculty of Humanities, University of Debrecen, Egyetem sqr. 1, Debrecen, 4032 Hungary

**Keywords:** Fundamental movement skills, Parental involvement, Motor skills, Physical activity

## Abstract

**Background:**

Encouraging physical activity and developing fundamental movement skills (FMS) is crucial for the healthy growth of children. Parental involvement can play a decisive role in shaping children’s physical activity habits; however, few studies have examined its direct impact on FMS. The aim of our study was to explore the extent to which parenting style, sporting habits, and the socio-cultural background of the family influence children’s movement skills.

**Methods:**

A cross-sectional study was conducted with the participation of 133 students (aged 10–13) from eight primary schools in Nyíregyháza, Hungary. The children’s fundamental movement skills were assessed using the KTK3 test, organized into three subtests: Walking Backwards (WB), Jumping Sideways (JS), and Moving Sideways (MS). Parental involvement and family background were assessed through a questionnaire. The study included several analyses of the data: the Mann-Whitney U and Kruskal-Wallis tests, as well as multivariate regression analysis.

**Results:**

The findings revealed that parental involvement was positively correlated with children’s WB performance (*p* < 0.05), while inconsistent discipline indicated a negative relationship with WB results (*p* < 0.05). The frequency of children’s physical activity was positively associated with FMS performance: individuals who engaged in sports several times a week achieved higher scores in balance and coordination tasks (*p* < 0.05). Furthermore, among parental sporting habits, fathers’ participation in recreational-level sports showed a positive association with children’s WB performance (*p* = 0.029). Modes of transportation were also found to shape results: regular car use and less frequent public transportation use were associated with better WB and MS outcomes (*p* < 0.05).

**Conclusions:**

Although the direct implication of parental support and parenting style was not evident in all cases, the findings suggest that parental involvement can enhance children’s movement skills, whereas inconsistent disciplinary practices may hinder their development. Strategies to support children’s motor skill progression should focus on the entire family’s lifestyle, with particular attention to sporting and transportation habits.

## Introduction

Parental beliefs, attitudes toward physical activity, the quality time parents spend with their children, and physical engagement all have a significant impact on child development, especially during the preschool years, which serve as the foundation for later life stages. Well-developed motor skills in childhood are positively associated with physical activity in adolescence and adulthood [1, 2]. Movement skills acquired during childhood play a meaningful role in shaping long-term physical activity. On the other hand, underdeveloped skills may hinder participation, since poor motor competence reduces self-confidence and motivation for physical activity, ultimately resulting in lower participation in sports and leisure-time physical activities [3].

Fundamental movement skills (FMS) thus not only form the basis of children’s everyday movement competence but are also of crucial importance for healthy development, commitment to physical activity, and long-term health status. The 10–12-year-old age group is considered a critical period for FMS, as these patterns become stabilized during this phase, yet further refinement and reinforcement are still possible [2]. International research suggests that parental support and parenting style during this developmental stage have considerable effects on children’s physical activity levels and motor competence [4].

Low physical activity levels and poor FMS can trigger a negative cycle gradually decreasing participation in physical activities and leading to a decline in motor skills and overall health. Consequently, children face a heightened risk of obesity-related conditions, such as type 2 diabetes and cardiovascular diseases [5]. Therefore, physical inactivity is a significant concern because it hinders motor skill progression and contributes to severe long-term health complications [6]. These considerations underscore the importance of prioritizing motor skill progression and parental support from an early age, especially in the area of FMS, given that these factors affect current health status, future quality of life, and even life expectancy [6].

According to the most recent findings of the Health Behaviour in School-aged Children (HBSC) international study, adolescent health behaviors are influenced by factors like parental support, the school environment, and peer relationships [7]. In Hungary, based on a representative 2022 survey of 11–15-year-old students using self-reported questionnaires, just over 20% meet the WHO’s recommendation of at least 60 min of moderate-to-vigorous physical activity (MVPA) per day, showing a strong correlation with perceived parental support [8]. At the same time, negative trends in mental health—such as increased stress and psychosomatic complaints—hinder commitment to regular physical activity [8].

These findings are in line with the objectives of our research, which aims to explore how different forms of parental involvement and the family’s socio-cultural background relate to children’s FMS. Parental involvement holds a meaningful potential for reversing negative trends. According to the theory of Epstein and Sanders [9], parental involvement refers to parents taking an active part in various aspects of their children’s life, including school performance, social relationships, and physical activity. A key element of this is joint participation in sports and movement-based activities, fostering positive movement patterns and increasing willingness to be active [10, 11]. In addition to supporting the child’s development, such experiences strengthen the relationship between parents and children.

Recent research also emphasizes that institutional practices promoting family-school-community partnerships have the potential to significantly enhance parental involvement across diverse socio-cultural contexts, for instance in majority and minority schools in Central and Eastern Europe [12]. The multifaceted nature of parental engagement is further highlighted by the correlation between parental involvement strategies and school choice and participation patterns in Hungarian primary schools [13]. Beyond academic contexts, teacher attitudes toward parental involvement (whether deficit-oriented or resource-focused) have also been found to greatly shape the effectiveness of family engagement practices [14].

Parental involvement is primarily studied in relation to academic achievement, and its function in health and motor skill attainment remains a research gap [15]. Previous studies have mostly focused on the role of parents’ socioeconomic status in health and motor skill progression, yet the combined analysis of multiple influencing factors is lacking, despite the multidimensional nature of FMS.

Our research intends to thoroughly investigate the factors influencing FMS among children between the ages of 10 and 13. We examine how parental involvement, parenting style, family sporting habits, transportation modes, and the socio-cultural background shape the FMS of students in this age group. While having practical relevance for health promotion initiatives, school physical education programs, and family-based health education, our results can contribute to laying the foundations of a healthy lifestyle.

## The role of family background and parental involvement in the development of movement skills

Parents’ attitudes toward physical activity play an essential part in the motor development of their children. Kimiecik and Horn [16] emphasize that experiences gained in the family environment are crucial for later physical activity. According to Li et al. [17], family participation and a supportive environment has a favourable impact on the advancement of children’s skills. Encouragement and feedback provided by parents can further strengthen children’s engagement in physical activity [18]. A systematic review found that parental support is positively associated with children’s physical activity, especially when parents are involved in various ways such as providing logistical support, encouragement, and joint participation in physical activities [19].

Parenting styles affect children’s activity levels in different ways. Permissive parenting increases screen time and authoritarian parenting decreases it [20]. As a systematic review suggests, authoritative and permissive parenting styles promote intrinsic motivation for physical activity, whereas neglectful and authoritarian approaches are less supportive [21]. A nurturing family environment, characterized by supportive parental attitudes and sibling relationships, is critical to children’s motor performance [22]. Parents who believe in the importance of physical exercise are generally more likely to raise physically active children [23].

Parents’ physical activity has strong implications for children’s motor skills: although previous research suggested that paternal activity might have a stronger impact on boys, while maternal activity determines girls’ motor skill progression more [24–26], recent findings indicate that gender differences between parent and child are less decisive. Recent systematic reviews have discovered a weak positive association between parental and child physical activity, regardless of gender [27]. Involving fathers in physical activity programs is one intervention that may prove particularly effective in increasing activity levels among girls [28]. All the above supports the notion that parental role modeling and support affect not only children’s physical activity but also their motor skill refinement and long-term attitudes toward sport, especially during the period when fundamental motor skills are acquired.

The ecological systems model developed by Bronfenbrenner [29] argues that multiple environmental factors shape child development. Family and school play a fundamental role in the formation of an active lifestyle [30]. The family’s socioeconomic status also determines the development of children’s movement skills. Previous studies have found that children whose parents have higher level education (college or university degree) typically perform better in motor competence tests than those whose parents have completed secondary, professional, or lower-level education [24].

The results presented clearly demonstrate that parents’ physical activity, parenting style, and the encouragement and logistical support they provide greatly contribute to the progression of children’s movement skills and their socialization toward regular physical activity. However, most of these studies concentrated on isolated aspects of parental influence and rarely examined the complexity of parental effects, for example the combined impact of different parenting characteristics, and often failed to consider socioeconomic background. The novelty of our study lies in the fact that it examines parenting styles, parental and child sports habits, and the prevalence of active and passive modes of transportation in addition to the importance of parental involvement. Socioeconomic background is analyzed as well, enabling a complex investigation into how parental involvement, parenting characteristics, and joint active leisure activities impact FMS, while controlling for background variables.

In Central and Eastern European countries, it is particularly important to map the factors affecting children’s physical activity, as physical inactivity remains a significant public health challenge in the region. Children’s healthy development depends heavily on the attainment of their motor skills and physical activity, aspects that have a lasting significance on their quality of life as adults [6].

The European Health Interview Survey (EHIS) reported that 59% of the adult population in Hungary does not engage in regular physical activity, which is slightly worse than the average for Central European countries and falls below the EU27 average [31]. Similarly, a 2021 regional report by the World Health Organization (WHO) indicated that only 42% of adolescents across the region met the daily MVPA recommendations. This figure reflects broader regional data and may include different age brackets and sampling methods compared to the HBSC findings [7, 32].

Based on these trends, it seems justified to examine the role of parents, family background, and sociodemographic factors in the motor development of children in this region, with special attention to the Hungarian context.

Parental support and involvement are key factors in children’s physical activity and motor skill acquisition. The objective of our research is to analyze how parenting style, sporting habits, and socio-cultural background contribute to children’s FMS. We formulated the following three main research questions:

1. How does parental involvement and parenting style influence children’s FMS?
2. What is the relationship between children’s sporting activity, transportation habits, and their FMS?
3. What role does the family’s socio-cultural background play in the development of FMS?

Based on the literature and the questions above, the following hypotheses were formulated:

H1: Parental involvement and a supportive parenting style have a positive impact on children’s FMS [22, 24, 27, 33].

H2: Children who engage in sports and use active modes of transportation show better FMS results [34–37].

H3: Children from more advantaged socioeconomic backgrounds have more developed movement skills [24, 38].

## Methodology

### Data collection and sample

Using stratified group sampling, the research was carried out as a cross-sectional study. To guarantee a sample with a range of socioeconomic backgrounds, the data collection took place in a major Hungarian city, involving eight randomly selected public and church-maintained schools. The research was carried out among 5th- and 6th-grade students aged 10 to 13 during May and June 2024. Data was gathered in two stages: first, permission was requested from the school principals, followed by the distribution of parental consent forms with the help of physical education teachers. During the second stage, the assessment was conducted, including an FMS test (KTK3) and a questionnaire filled out by students. Although 155 students initially participated in the survey, data from 22 participants were excluded due to incomplete responses or missing data, resulting in a final analytical sample of 133 students.

### Participants

The characteristics of the participants are summarized in the table below (Table 1).

**Table 1:** Sociocultural and demographic characteristics of the sample (*N* = 133)

Variable	Value	%	*N*
Age	10	9.8	13
	11	32.3	43
	12	40.6	54
	13	17.3	23
Grade	5	55.6	74
	6	44.4	59
School type	Public	80.5	107
	Church-maintained	19.5	26
What is your mother’s/stepmother’s highest level of education?	Primary school	13.8	18
	Secondary school	19.2	25
	Vocational school	16.9	22
	College or university	50.0	65
What is your father’s/stepfather’s highest level of education?	Primary school	17.7	23
	Secondary school	16.2	21
	Vocational school	22.3	29
	College or university	43.8	57
How would you describe your family’s financial situation?	We have everything we need, can afford major expenses, and save money	65.2	86
	We have everything we need, but cannot afford major expenses	27.3	36
	Sometimes we cannot cover our daily expenses	5.3	7
	We are often unable to cover our basic needs	2.3	3

**Source**: author’s own compilation

### Measurements and analysis

Children’s motor skills were measured using the shortened version of the KTK4 test designed by Kiphard and Schilling, known as KTK3 [39]. The KTK4 is one of the most internationally validated, and widely used tools for assessing motor coordination and fundamental movement skills in children [40, 41]. In addition, it shows good correlations with other motor assessment tools and is frequently used as a reliable reference in comparative validation studies [40]. The test includes three of the original four tasks: Walking Backwards (WB), Jumping Sideways (JS), and Moving Sideways (MS), as the fourth task (Hopping for Height– HH) was excluded due to time constraints in the schools’ schedule [42].

In the JS task, participants had to laterally jump over a wooden slat within 15 s, performed twice. The MS task involved stepping from one floor plate to the next and moving the previous plate within 20 s, also performed twice. The WB task was conducted on balance beams of three different widths, with three attempts per beam. Measurements were carried out by the researcher and trained assistants experienced in administering the tests. For data analysis, the best performance of each participant was considered.

To determine the background factors of FMS, the students also completed a paper-based questionnaire after the test assessment. Our questionnaire contained 59 items overall, with 4 open-ended questions (name of the school, grade, age, number of siblings) and 55 closed-ended items covering six dimensions.

In order to assess parental support, a modified version of the *M-PAC Applied to Parental Support of Child Physical Activity* questionnaire was used [43], which comprised six items (Cronbach’s α = 0.616, KMO = 0.739, *p* < 0.001, explained variance: 52.8%).

To assess parenting styles, a modified version of the *Alabama Parenting Questionnaire (APQ)* was applied [44], consisting of 30 items which were evaluated across four factors: positive parenting, involvement, poor monitoring, and inconsistent discipline (Cronbach’s α = 0.675, KMO = 0.822, *p* < 0.001, explained variance: 42.9%).

The measurement instruments used in the study, along with their main psychometric characteristics, are summarized in Table 2.

**Table 2:** Measurement instruments and reliability statistics used in the study

Instrument	Dimension measured	No. of items	Cronbach’s α	KMO	Explained variance	Source
KTK3 Test	Fundamental movement skills (WB, JS, MS)	3 tasks	–	–	–	Lovell (2017) [39]
M-PAC questionnaire (modified)	Parental support for physical activity	6	0.616	0.739	52.8%	M-PAC– University of Victoria [43]
Alabama Parenting Questionnaire (modified)	Parenting style (positive parenting, involvement, poor monitoring, inconsistent discipline)	30	0.675	0.822	42.9%	Zlomke et al. (2015) [44]

**Source**: author’s own compilation

Additional sections of the questionnaire assessed various lifestyle and background factors. Parental physical activity was examined through questions addressing both current and past sport participation of the mother and father, including their level of involvement (e.g., recreational, amateur, competitive, or non-participant) and the type of sport practiced (e.g., ball games, martial arts, athletics). Further items covered children’s physical activity (frequency and parental role modeling), transportation habits (e.g., car, bicycle, public transport, walking), and sociocultural and demographic background, such as gender, age, parents’ education level, type of residential settlement, financial situation, and number of siblings.

For data processing, the SPSS 29 statistical software was used. Analyses included principal component analysis, factor analysis, cluster and regression analyses, as well as Mann–Whitney U and Kruskal–Wallis tests. The normality of continuous variables was checked by using the Kolmogorov–Smirnov test.

## Results

The findings highlight the critical role of parental support in the development of children’s movement skills. Among the respondents, 63.9% reported receiving regular encouragement to engage in physical activity, with 41.4% exercising outdoors joint physical activity with parents every day and another 30.8% on most days. This suggests that over 70% of children regularly participate in physical activity, which is a positive factor for healthy development.

**Fig. 1:**
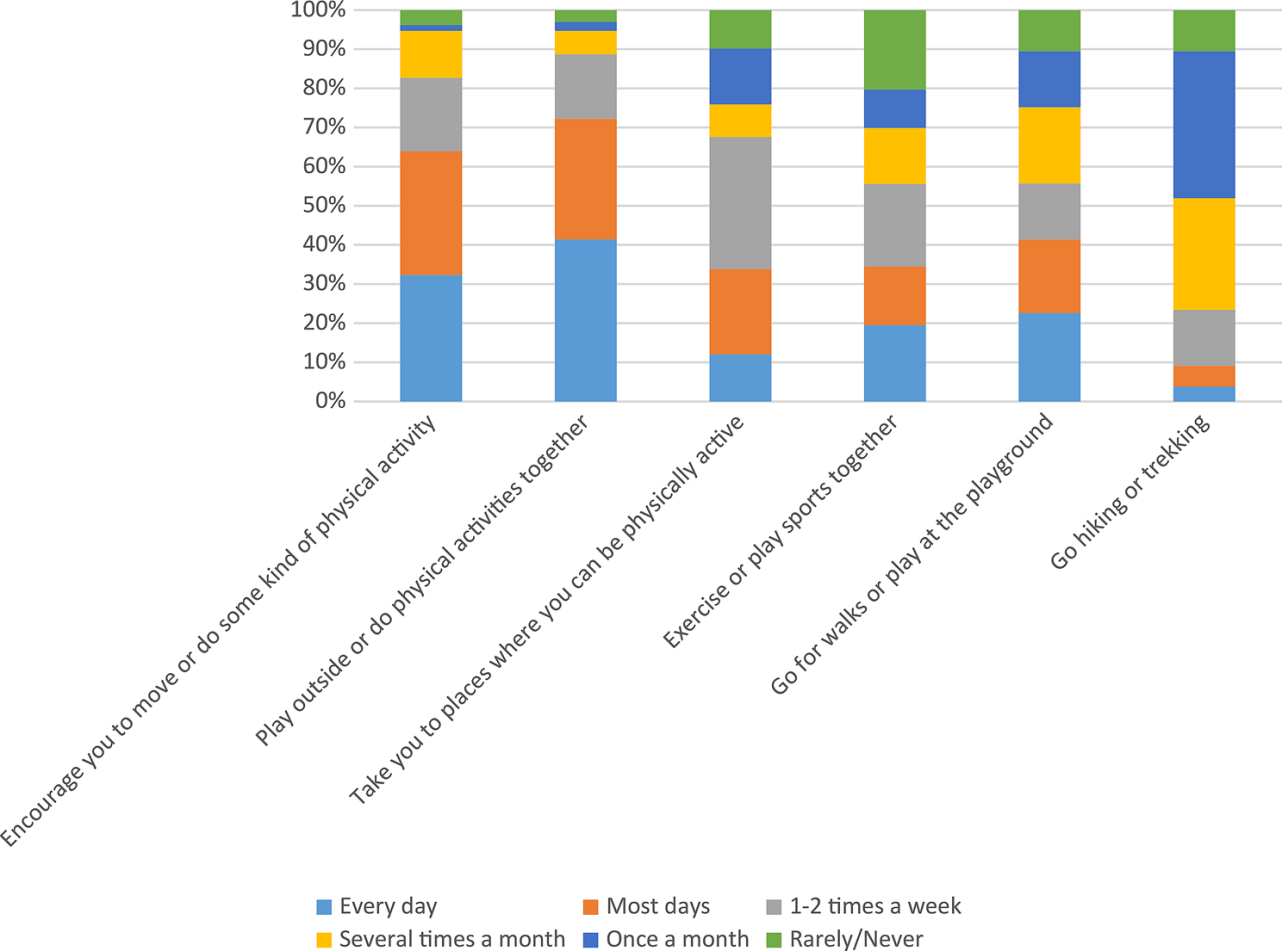
Basic distribution of responses to questions about parental support (*N* = 133). Source: own research

Parents’ active involvement in their children’s physical activities is also apparent: 33.8% of them take their children to sporting activities 1–2 times per week, 21.8% do so more frequently, and 12% provide such opportunities on a daily basis. Joint physical activity is also common, with 21.1% exercising with family members 1–2 times per week and 19.5% doing so daily (Fig. 1).

No significant correlations or relationships were found between parental support, parenting styles, and FMS based on either correlation or bivariate analyses (both with original and dichotomized variables). The relationships were analyzed across the three test subcomponents and the total FMS score, but no statistically significant associations were observed.

The analysis of parental sporting habits revealed that 36.1% of fathers had participated in ball sports, but only 15.8% of mothers reported the same. Among fathers, 7.5% currently participate in competitive sports and 39.1% engage in sports recreationally. Among mothers, 9% participate competitively and 30.1% at a recreational level.

According to the Kruskal–Wallis test, the level of the father’s sporting activity was significantly associated with children’s WB performance. Children whose fathers played recreational-level sports performed better, while those with competitive athlete fathers had lower scores (Table 3).

Regular participation in sports fostered improvements in movement skills. Children who did sports 1–2 times per week had lower scores than those who exercised more frequently. Notably, children who engaged in physical activity three or more times per week achieved higher scores in JS, MS, and total FMS compared to those who were less active (Table 3).

The analysis of transportation habits revealed that the frequency of car uses significantly affected performance in the WB and MS tests. Active forms of transportation (e.g., walking, cycling) had a positive relationship with FMS. Public transportation usage also influenced movement skills. Children who used public transport less frequently performed better on the WB and MS tests. This may suggest that a more active lifestyle has a beneficial effect on motor skills (Table 3).

Performance on the MS test differed significantly between 5th and 6th grade students, with 5th graders outperforming their 6th-grade peers, possibly indicating a different developmental pace. Gender differences were also significant in the JS test and the overall FMS score. Boys performed better in the JS test than girls and also showed higher overall motor skill scores. Rural students achieved higher average scores in total FMS compared to urban students (Table 3). It is important to clarify that all participating schools were in an urban environment. The distinction between rural and urban students was based on the type of residential settlement reported by the participants (e.g., village, town, or city), rather than the location of the schools themselves.

**Table 3:** Summary of statistically significant differences in fundamental movement skills based on background variables

Variable	FMS Dimension	Group Comparison (Mean ± SD)	Test Statistic	*p*-value	*N*
*Father’s sport participation*	WB	Amateur (competition): 21.17 ± 3.60; Amateur (recreational): 18.67 ± 3.62; Competitive: 18.00 ± 3.39; Non-sporting: 20.03 ± 4.31	H = 10.776	0.029	105
*Child’s sport frequency*	JS	< 1x/week: 26.24 ± 6.21; 1x/week: 30.63 ± 6.27; ≥3x/week: 31.82 ± 5.86	H = 11.427	0.010	130
	MS	< 1x/week: 6.76 ± 1.70; 1x/week: 7.92 ± 1.92; ≥3x/week: 7.85 ± 2.10	H = 6.114	0.047	131
	FMS Total	< 1x/week: 51.00 ± 9.14; 1x/week: 58.39 ± 9.96; ≥3x/week: 59.30 ± 8.34	H = 12.380	0.006	130
*Car use frequency*	WB	Never: 18.74 ± 4.44; Sometimes/year: 18.92 ± 3.80; Monthly: 19.33 ± 4.36; Weekly: 20.50 ± 2.11; Daily: 19.56 ± 4.01	H = 11.307	0.023	133
	MS	Never: 7.19 ± 1.84; Sometimes/year: 6.85 ± 1.82; Monthly: 8.00 ± 2.35; Weekly: 7.25 ± 1.86; Daily: 8.04 ± 2.04	H = 11.183	0.025	133
*Public transport use*	WB	Never: 19.91 ± 3.47; Sometimes/year: 17.91 ± 4.36; Monthly: 21.06 ± 3.77; Weekly: 17.78 ± 3.24; Daily: 19.43 ± 4.62	H = 11.307	0.023	133
	MS	Never: 8.05 ± 2.01; Sometimes/year: 6.48 ± 1.24; Monthly: 8.00 ± 2.22; Weekly: 7.50 ± 1.99; Daily: 7.83 ± 2.17	H = 11.183	0.025	133
*Walking frequency*	JS	Never: 32.41 ± 5.24; Sometimes/year: 30.67 ± 9.34; Monthly: 29.50 ± 4.00; Weekly: 30.85 ± 6.45; Daily: 28.49 ± 6.16	H = 9.988	0.041	132
*Grade level*	MS	5th grade: 8.09 ± 2.10; 6th grade: 7.15 ± 1.77	U = 1612.000; Z = − 2.617	0.009	133
*Gender*	JS	Boys: 31.81 ± 6.27; Girls: 28.30 ± 5.63	Z = − 3.392	< 0.001	132
	FMS Total	Boys: 59.01 ± 9.38; Girls: 55.30 ± 8.78	Z = − 2.288	0.022	132
*Type of residence (5 levels)*	FMS Total	Farm: 65.25 ± 5.90; Large city: 58.60 ± 9.02; Mid-sized city: 55.32 ± 9.96; Small town: 56.21 ± 7.99; Village: 59.55 ± 9.38	H = 10.410	0.034	131
*Urban vs. rural area*	FMS Total	Rural: 61.18 ± 8.82; Urban: 56.76 ± 9.30	U = 1032.5	0.021	131

**Source**: author’s own compilation

**Notes.** The means and standard deviations (SD) presented in the table refer to the highest and lowest scoring subgroups within each variable category. Only statistically significant group differences (*p* < 0.05) are included. The test statistics are reported as follows: H indicates the Kruskal–Wallis H test used for comparing three or more independent groups; U refers to the Mann–Whitney U test for two-group comparisons; W denotes the Wilcoxon rank-sum value; and Z represents the standardized test statistic associated with the Mann–Whitney U test

The aim of the multivariate analysis was to identify which factors influence children’s FMS. Three multistep linear regression models were applied. The first model examined parental involvement and parenting styles; the second added the sporting habits not only of the child, but that of the parents, too, and the transportation forms; the third model included socioeconomic background variables.

For the WB variable, the second model proved to be the strongest: inconsistent discipline had a negative effect (β = − 0.319; *p* = 0.024), whereas positive parenting (β = 0.351; *p* = 0.026), parental involvement (β = 0.393; *p* = 0.008), and the child’s frequency of physical activity (β = 0.402; *p* = 0.007) emerged as positive predictors (*R²* = 0.390; *p* = 0.047). The third model confirmed the role of involvement (β = 0.373; *p* = 0.018), frequency of activity (β = 0.328; *p* = 0.046), and positive parenting (β = 0.327; *p* = 0.049), although the model itself was not statistically significant (*R²* = 0.462; *p* = 0.141).

For the JS variable, the second model indicated significant associations (*R²* = 0.442; *p* = 0.013), where frequency of physical activity (β = 0.320; *p* = 0.023), the father’s past sport activity (β = − 0.359; *p* = 0.038), the mother’s past sport activity (β = 0.544; *p* = 0.004), and bicycle use (β = 0.268; *p* = 0.048) influenced the results. The third model (*R²* = 0.534; *p* = 0.032) confirmed the significant effect of the mother’s past sport activity (β = 0.513; *p* = 0.011).

For the MS subdimension, the second model implied a significant positive association with car use (β = 1.859; *p* = 0.007), while the third model demonstrated negative effects of the mother’s current physical activity (β = − 1.477; *p* = 0.036) and the family’s financial situation (β = − 1.389; *p* = 0.028).

Regarding the overall fundamental movement score, only the frequency of sport participation emerged as a significant predictor in the second model (β = 0.403; *p* = 0.001; *R²* = 0.365). Despite the third model increasing explanatory power (*R²* = 0.462), it did not yield any additional significant predictors.

## Discussion

The aim of this study was to explore the factors influencing the development of fundamental movement skills (FMS), with particular attention to parental involvement and parenting styles, sport participation and modes of transportation, as well as the contribution of socioeconomic background. Based on these three core areas, we formulated hypotheses to gain a comprehensive picture of the factors that may support or hinder the progression of children’s motor skills.

The first key research question addressed how parental involvement, parenting styles, and parents’ sporting habits affect children’s movement skills. The results indicated that positive parenting and involvement had a favorable effect on the WB score, but no clear relationship was found with other FMS indicators. This suggests that parental involvement may primarily influence balance and coordination skills, yet strength and speed-related components seem to be shaped by other factors. Based on this, our first hypothesis was partially confirmed.

The findings are consistent with previous research. Flynn et al. [33] and Derikx et al. [22] demonstrated that parental support and involvement foster motor development in children, especially in fine motor coordination. According to Kimiecik and Horn [16], parenting style helps to determine a child’s attitude toward physical activity and impacts parent-child communication, thereby contributing to motor competence.

Inconsistent discipline was found to have a negative effect on the WB score. This implies that not only the presence of parental support but also its quality is crucial for a child’s development. Earlier studies have confirmed that authoritative parenting supports motor skill progression, whereas inconsistent or overly controlling styles may inhibit it [45].

The mother’s past participation in sports was found to have a positive impact on JS performance. In contrast, no evident link was identified between the father’s sporting history and movement skills. This may suggest that the mother’s sporting example has a more lasting implication on children’s motor development. At the same time, the mother’s current physical activity emerged as a negative predictor in the MS test. This is in line with the findings of Danioni et al. [46], who argued that maternal pressure can sometimes negatively influence children’s performance, particularly when parental expectations are too high. Beyond parental pressure, other contextual factors may also contribute to this finding. For example, children may perceive frequent maternal activity as a source of implicit comparison or expectation, which could impact their confidence or motivation [46, 47]. It is also possible that highly active mothers have less time to engage directly with their children’s physical development, or that their activity routines are not shared experiences, reducing the effectiveness of role modeling [48].

The second research question addressed the relationship between children’s frequency of sport participation and transportation modes and their FMS performance. This hypothesis was confirmed, since the results implied that regular sport participation has a positive influence on the attainment of motor skills. A total of 41.4% of respondents reported engaging in daily physical activity with parents, which closely aligns with the 42% reported in the WHO’s 2021 regional data on Hungarian adolescents’ daily MVPA [32]. Additionally, 53.8% of children reported engaging in sport at least three times per week outside of PE classes. These favorable activity levels may partly be explained by the characteristics of the sample and the urban setting in which the study was conducted. The city’s infrastructure, school-based programs, and parental support may all contribute to more frequent engagement in physical activity compared to national trends.

Sport activities outside of physical education classes were significant predictors for both WB and JS performance, which supports the importance of physical activity in establishing motor skills. However, it is important to note that the relationship may also be bidirectional: children with more advanced motor skills may approach sports with greater self-confidence and perceived competence, thereby enhancing their participation in physical activity [1, 3].

The results concerning transportation habits were mixed. Cycling had a positive effect on JS performance, while the frequency of car use was related to WB and MS scores. Although walking frequency was not found to have a clear beneficial impact, children who walked less often performed better in the JS test. This finding contradicts previous expectations and suggests that the outcomes of transportation habits may be context-dependent. It is possible that socioeconomic status, infrastructure, and parental transportation patterns all shape this relationship.

Our third research question explored the extent to which socioeconomic background defines children’s motor skills. Contrary to our hypothesis, this relationship was not confirmed. Our analysis revealed that the family’s financial situation has no notable connection to FMS. This contradicts previous research, such as the study by Cools et al. [24]. They found a significant positive relationship between parents’ educational attainment and children’s motor development. Maric et al. [38] concluded that parents with higher educational backgrounds are more likely to encourage their children to be physically active. The results imply that FMS development is more strongly related to parental involvement and sport habits than to social status alone.

It is important to note that even though the direct impact of socioeconomic status on FMS was not significant in this study, indirect impacts could still exist, potentionally nuancing the interpretation. Background factors like parental education or financial situation, may influence parenting styles, the level of parental involvement, and children’s access to sport opportunities. These factors, in turn, directly affect the establishment of motor skills. A comparison of the determinants of physical activity found in the literature with those examined in the study showed in Table 4.

**Table 4:** Comparison of predictors of children’s FMS and motor competence: Findings between the literature and the present study

Factor	Literature Finding	Result in this study	FMS Dimension(s) Affected	Key Literature Sources
*Parental Support*	Positive impact on PA and motor skills	Significant positive association with WB performance	WB	Flynn et al. (2023) [33]; Rhodes et al. (2020) [19]
*Positive Parenting Style*	Enhances FMS and intrinsic motivation	Positively predicted WB score in regression models	WB	Kimiecik & Horn (2012) [16]; Danioni et al. (2017) [46]
*Parental Involvement*	Encourages skill development, value transmission	Strongest positive predictor of the WB performance	WB	Flynn et al. (2023) [33]; Danioni et al. (2017) [46]
*Inconsistent Discipline*	Often undermines autonomy and skill progression	Significant negative predictor of WB performance	WB	Kimiecik & Horn (2012) [16]
*Parental Sport Participation– Mother (Current)*	Mixed; may lead to pressure or modeling effects	Negatively predicted MS score	MS	Danioni et al. (2017) [46]
*Parental Sport Participation– Mother (Past)*	Positive modeling, latent value transmission	Positively associated with JS performance	JS	Cools et al. (2011) [24]; Moore et al. (1991) [25]
*Parental Sport Participation– Father (Past)*	Inconsistent impact in literature, but trend of positive influence	No significant association with any FMS dimension	–	Moore et al. (1991) [25]
*Parental Sport Participation– Father (Current)*	More activity linked with greater FMS in children	Recreational-level activity linked to higher WB scores	WB	Moore et al. (1991) [25]
*Child’s Sport Frequency*	Clear predictor of FMS	Frequent activity (≥ 3x/week) predicted higher JS, MS, and Total FMS	JS, MS, Total	Flynn et al. (2023) [33]; Maric et al. (2020) [38]
*Car Use Frequency*	Literature: mostly negative association with FMS	More frequent car use linked to better WB and MS performance	WB, MS	Stark et al. (2024) [36]; Zhu et al. (2024) [37]
*Public Transport Use*	Associated with lower motor skill levels	Less frequent use associated with better WB and MS scores	WB, MS	Lubans et al. (2011) [35]; Stark et al. (2024) [36]; Zhu et al. (2024) [37]
*Walking/Cycling Frequency*	Positive association with FMS and coordination	Daily walkers showed lower JS scores; effect context-dependent	JS	Lubans et al. (2011) [35]; Stark et al. (2024) [36]; Zhu et al. (2024) [37]
*Grade / Age*	Mixed in lit.; often younger = weaker FMS	5th graders outperformed 6th graders in MS	MS	Cools et al. (2011) [24]; Derikx et al. (2021) [22]
*Gender*	Boys often perform better in object control & gross skills	Boys scored higher in JS and Total FMS	JS, Total	Flynn et al. (2023) [33]; Moore et al. (1991) [25]
*Urban–Rural Residence*	Rural settings may support informal activity	Rural residents had higher average Total FMS scores	Total	Cools et al. (2011) [24]; Stark et al. (2024) [36]
*Parental Education (SES)*	Higher SES often linked with better PA and FMS	No clear association with any FMS outcome	–	Cools et al. (2011) [24]; Maric et al. (2020) [38]

**Source**: author’s own compilation

This study has several limitations that must be considered when interpreting the results. Due to the cross-sectional research design, causal relationships cannot be established—only correlations. Data collection was based on self-reported questionnaires, which might have introduced bias due to the participants’ subjective perceptions. The sample size and composition limit the generalizability of the findings, especially since the study focused on a specific geographic region. Because of this, the results cannot be generalized to the entire national population, due to potential regional differences in socioeconomic status, infrastructure, and lifestyle habits. FMS was assessed using the shortened KTK3 version, which may not fully reflect children’s overall motor development. Finally, a more in-depth investigation of socioeconomic and family background effects would require qualitative research methods. These limitations suggest that future research should use longitudinal designs with larger, more diverse samples, and combine objective measurement tools with self-reports.

At the same time, it is important to highlight that our research was able to examine the role of factors presented in the literature in a multidimensional way, controlling for their mutual implications. This contributes to the international discourse on the contradictory findings regarding the effects of parental involvement, parenting, sport, and family socioeconomic background. Moreover, our study was conducted in a specific, disadvantaged region where there is a high proportion of children who are overweight or obese and in need of motor development support.

## Conclusion

The main finding of our research is that parental involvement and parenting styles significantly influence the development of children’s fundamental movement skills. Supportive parental behavior positively affects motor performance, while inconsistent discipline has a negative impact. The parents’ physical activity showed mixed results: past sport participation had a stronger influence than current activity, suggesting that parental attitudes may have long-term effects on children’s motor development.

Based on our results, physical education programs and health promotion strategies can be enhanced by encouraging parental involvement and children’s sport participation. Targeted programs can help support disadvantaged students, and promoting leisure-time sport can reduce the risks associated with a sedentary lifestyle. Future research should further explore these relationships to gain a deeper understanding, particularly regarding the differing roles of family members and the effects of various socioeconomic factors on children’s physical development. Additional research directions could also include the availability of sport opportunities for children, as well as examining the content of physical education lessons and children’s attitudes toward them.

## Data Availability

The data that support the findings of this study are available from the corresponding authors, but restrictions apply to their availability. These were used under license for the current study, and, so, are not accessible by the public. Data are, however, available from the corresponding authors upon reasonable request and with permission of the School Ethics Committee of Doctoral Program on Educational Sciences at the University of Debrecen.

## Abbreviations

FMS: Fundamental movement skills
WB: Walking backwards
JS: Jumping sideways
MS: Moving sideways
HH: Hopping for height
HBSC: Health behaviour in school-aged children
EHIS: European health interview survey
WHO: World health organization

## References

[1] Barnett LM, van Beurden E, Morgan PJ, Brooks LO, Beard JR. Does childhood motor skill proficiency predict adolescent fitness? Med Sci Sports Exerc. 2008;40(12):2137–44. 10.1249/MSS.0b013e31818160d3. 18981934 10.1249/MSS.0b013e31818160d3

[2] Stodden DF, Goodway JD, Langendorfer SJ, Roberton MA, Rudisill ME, Garcia C, et al. A developmental perspective on the role of motor skill competence in physical activity: an emergent relationship. Quest. 2008;60(2):290–306. 10.1080/00336297.2008.10483582.

[3] Allender S, Cowburn G, Foster C. Understanding participation in sport and physical activity among children and adults: A review of qualitative studies. Health Educ Res. 2006;21(6):826–35. 10.1093/her/cyl063. 16857780 10.1093/her/cyl063

[4] Jago R, Davison KK, Brockman R, Page AS, Thompson JL, Fox KR. Parenting styles, parenting practices, and physical activity in 10- to 11-year-olds. Prev Med. 2011;52(1):44–7. 10.1016/j.ypmed.2010.11.001. 21070805 10.1016/j.ypmed.2010.11.001 PMC3025352

[5] Cliff DP, Okely AD, Morgan PJ, Jones RA, Steele JR, Baur LA. Proficiency deficiency: mastery of fundamental movement skills and skill components in overweight and obese children. Obes (Silver Spring). 2012;20(5):1024–33. 10.1038/oby.2011.241. 10.1038/oby.2011.241 21799480

[6] World Health Organization. Global status report on physical activity 2022. Geneva, WHO. 2022. Licence: CC BY-NC-SA 3.0 IGO. ISBN: 978-92-4-005915-3. [cited 2025 Mar 28]. Available from: https://www.who.int/publications/i/item/9789240059153

[7] Badura P, Eriksson C, García-Moya I, Löfstedt P, Melkumova M, Sotiroska K et al. The Health Behaviour in School-aged Children (HBSC) study. Geneva: World Health Organization; 2024. Report No.: Volume 7. ISBN: 9789289061391.

[8] Németh Á. szerk. Iskoláskorú gyermekek egészségmagatartása 2022: A WHO-val együttműködésben megvalósuló nemzetközi kutatás nemzeti jelentése– legújabb adatok és trendek az elmúlt húsz évben [Health Behaviour in School-Aged Children, 2022. Health Behaviour in School-aged Children (HBSC) A WHO-collaborative Cross-National Study 2022 National Report– newest data and trends from the last twenty years]. Budapest: L’Harmattan; ELTE Faculty od Pedagogy and Psychology; 2024. Hungarian. 10.56037/978-963-646-075-4

[9] Epstein JL, Sanders MG. School, family, and community partnerships. In: Bornstein MH, editor. Handbook of parenting: practical issues in parenting. Volume 5. Mahwah, NJ: Lawrence Erlbaum Associates; 2002. pp. 407–38.

[10] Hamilton M. Parent-assisted instruction in a motor skill program for at-risk preschool children. Adapt Phys Activ Q. 1999;16:415–26.

[11] Rhodes RE, Guerrero MD, Vanderloo LM, Tremblay MS, Williams G. Development of a consensus statement on the role of the family in the physical activity, sedentary, and sleep behaviours of children and youth. Int J Behav Nutr Phys Act. 2020;17:74. 10.1186/s12966-020-00973-0. 32539730 10.1186/s12966-020-00973-0 PMC7296673

[12] Pusztai G, Bacskai K, Ceglédi T, Kocsis Z, Hine MG. Mission possible? Institutional Family-School-Community partnership practices and parental involvement in Hungarian majority and minority schools in three central and Eastern European countries. Social Sci. 2025;14(2):107.

[13] Pusztai G, Róbert P, Fényes H. Parental involvement and school choice in Hungarian primary schools. J School Choice. 2023;17(1):118–35.

[14] Pusztai G, Demeter-Karászi Z, Major E, Puskás M. Beyond the barriers of deficit orientedness? Comparing distinct teacher approaches of parental involvement. Br J Religious Educ. 2024;46(4):370–88.

[15] Kovács K, Oláh ÁJ, Pusztai G. The role of parental involvement in academic and sports achievement. Heliyon. 2024;10(2):e24290. 10.1016/j.heliyon.2024.e24290. 38293479 10.1016/j.heliyon.2024.e24290 PMC10824762

[16] Kimiecik JC, Horn TS. Examining the relationship between family context and children’s physical activity beliefs: the role of parenting style. Psychol Sport Exerc. 2012;13(1):10–8. 10.1016/j.psychsport.2011.08.004.

[17] Li J, Yan Y, Yin T. Does parental involvement contribute to students’ development? The parent-child homework experiment at a Shanghai migrant school. Int J about Parents Educ. 2015;9(1):1–9.

[18] Ornelas IJ, Perreira KM, Ayala GX. Parental influences on adolescent physical activity: A longitudinal study. Int J Behav Nutr Phys Act. 2007;4(1):3. 10.1186/1479-5868-4-3. 17274822 10.1186/1479-5868-4-3 PMC1805507

[19] Rhodes RE, Perdew M, Malli S. Correlates of parental support of child and youth physical activity: a systematic review. Int J Behav Med. 2020;27(6):636–46. 10.1007/s12529-020-09909-1. 32529629 10.1007/s12529-020-09909-1

[20] Van der Geest KE, Mérelle SYM, Rodenburg G, Van de Mheen D, Renders CM. Cross-sectional associations between maternal parenting styles, physical activity, and screen sedentary time in children. BMC Public Health. 2017;17:1–11. 28962600 10.1186/s12889-017-4784-8 PMC5622508

[21] Vega-Díaz R, Pérez-Turpin JA, Falcó C, García-Romero C, Tárraga-López PJ. Parenting profiles: motivation toward health-oriented physical activity and intention to be physically active. BMC Psychol. 2023;11:239. 10.1186/s40359-023-01239-7. 37438804 10.1186/s40359-023-01239-7 PMC10339519

[22] Derikx DFAAA, Houwen S, Meijers V, Schoemaker MM, Hartman E. The relationship between social environmental factors and motor performance in 3- to 12-year-old typically developing children: A systematic review. Int J Environ Res Public Health. 2021;18(14):7516. 10.3390/ijerph18147516. 34299967 10.3390/ijerph18147516 PMC8306533

[23] Barnett LM, Hnatiuk JA, Salmon J, et al. Modifiable factors which predict children’s gross motor competence: A prospective cohort study. Int J Behav Nutr Phys Act. 2019;16:129. 10.1186/s12966-019-0888-0. 31829267 10.1186/s12966-019-0888-0 PMC6907285

[24] Cools W, De Martelaer K, Samaey C, Andries C. Fundamental movement skill performance of preschool children in relation to family context. J Sports Sci. 2011;29(7):649–60. 21424981 10.1080/02640414.2010.551540

[25] Moore LL, Lombardi DA, White MJ, Campbell JL, Oliveria SA, Ellison RC. Influence of parents’ physical activity levels on activity levels of young children. J Pediatr. 1991;118(2):215–9. 1993947 10.1016/s0022-3476(05)80485-8

[26] Fuemmeler BF, Anderson CB, Mâsse LC. Parent-child relationship of directly measured physical activity. Int J Behav Nutr Phys Act. 2011;8:17. 10.1186/1479-5868-8-17. 21385455 10.1186/1479-5868-8-17 PMC3062578

[27] Petersen TL, Mavoa H, Faulkner G, Hinkley T. Association between parent and child physical activity: a systematic review. Int J Behav Nutr Phys Act. 2020;17:74. 10.1186/s12966-020-00966-z. 32423407 10.1186/s12966-020-00966-z PMC7236180

[28] Morgan PJ, Young MD, Barnes AT, Eather N, Pollock ER, Lubans DR. Engaging fathers to increase physical activity in girls: the ‘dads and daughters exercising and empowered’ (DADEE) randomized controlled trial. Int J Behav Nutr Phys Act. 2019;16(1):67. 10.1093/abm/kay015. 29648571 10.1093/abm/kay015

[29] Bronfenbrenner U. The ecology of human development: experiments by nature and design. Cambridge, MA: Harvard University Press; 1979.

[30] Ferreira I, van der Horst K, Wendel-Vos W, Kremers S, van Lenthe FJ, Brug J. Environmental correlates of physical activity in youth: A review and update. Obes Rev. 2007;8(2):129–54. 10.1111/j.1467-789X.2006.00264.x. 17300279 10.1111/j.1467-789X.2006.00264.x

[31] Hungarian Central Statistical Office (KSH). European Health Interview Survey (EHIS) 2019– Physical activity indicators. Budapest: KSH. 2019 [cited 2025 Mar 28]. Available from: https://www.ksh.hu/ehis_timeseries

[32] World Health Organization. Physical Activity Factsheet– Hungary. (2021). WHO Regional Office for Europe; 2021 [cited 2025 Mar 28]. Available from: https://www.who.int/europe/publications/m/item/physical-activity-factsheet-hungary-2021

[33] Flynn RJ, Pringle A, Roscoe CMP. Direct parent engagement to improve fundamental movement skills in children: A systematic review. Child (Basel). 2023;10(7):1247. 10.3390/children10071247. 10.3390/children10071247 PMC10378247 37508744

[34] Barros P, Guerra PH, Khan M, Fermino RC. Impact of active travel to school on children’s health: an overview of systematic reviews. Transp Res Procedia. 2024;76:156–68. 10.1016/bs.atpp.2023.11.003.

[35] Lubans DR, Boreham CA, Kelly P, Foster CE. The relationship between active travel to school and health-related fitness in children and adolescents: A systematic review. Int J Behav Nutr Phys Act. 2011;8:5. 10.1186/1479-5868-8-5. 21269514 10.1186/1479-5868-8-5 PMC3039551

[36] Stark J, Faber F, Wulfhorst G. Activities and active mobility of children– At the interface of travel behavior and health research. Transp Res Procedia. 2023;76:225–37. 10.1016/j.trpro.2023.12.046.

[37] Zhu X, Wang X, Li Y. Understanding children’s active school commute, independent mobility, and physical activity in Austin, Texas, USA: roles of physical environments. Front Archit Res. 2024;13(2):254–67. 10.1016/j.foar.2024.02.016.

[38] Maric D, Kvesic I, Lujan IK, Bianco A, Zenic N, Separovic V, et al. Parental and Familial factors influencing physical activity levels in early adolescence: A prospective study. Healthc (Basel). 2020;8(4):532. 10.3390/healthcare8040532. 10.3390/healthcare8040532 PMC7761556 33276633

[39] Lovell T. Factors affecting engagement and talent development in a school-based sports program [dissertation]. Sydney: University of Technology Sydney; 2017. https://opus.lib.uts.edu.au/bitstream/10453/123182/7/02whole.pdf

[40] Cools W, De Martelaer K, Samaey C, Andries C. Movement skill assessment of typically developing preschool children: A review of seven movement skill assessment tools. J Sports Sci Med. 2009;8(2):154–68. 24149522 PMC3761481

[41] Vandorpe B, Vandendriessche J, Lefevre J, Pion J, Vaeyens R, Matthys S, et al. The Körperkoordinationstest für Kinder: Reference values and suitability for 6–12-year-old children in Flanders. Scand J Med Sci Sports. 2011;21(3):378–88. 10.1111/j.1600-0838.2009.01067.x. 20136753 10.1111/j.1600-0838.2009.01067.x

[42] Biino V, Giustino V, Guidetti L, Lanza M, Gallotta M, Baldari C, et al. Körperkoordinations test für Kinder: A short form is not fully satisfactory. Front Educ. 2022;7. 10.3389/feduc.2022.914445.

[43] University of Victoria. M-PAC Applied to Parental Support of Child Physical Activity– Sample Questionnaire [Internet]. [cited 2024 May 13]. Available from: https://onlineacademiccommunity.uvic.ca/mpac/wp-content/uploads/sites/3020/2018/11/M-PAC-Questionnaire-Applied-to-Parental-Support-for-Child-PA.pdf

[44] Zlomke K, Bauman S, Lamport D. Adolescents’ perceptions of parenting behavior: Validation of the Alabama parenting questionnaire adolescent self-report. J Child Fam Stud. 2015;24:2631–40. 10.1007/s10826-015-0119-5.

[45] Yaffe Y. Physical activity among Israeli-Arab adolescent males: how do parenting styles matter? Am J Mens Health. 2018;12(6):2037–43. 10.1177/1557988318790881. 30043663 10.1177/1557988318790881 PMC6199442

[46] Danioni F, Barni D, Rosnati R. Transmitting sport values: the importance of parental involvement in children’s sport activity. Eur J Dev Psychol. 2017;14(2):192–205. https://ejop.psychopen.eu/index.php/ejop/article/view/1265. 10.5964/ejop.v13i1.1265 PMC5342312 28344676

[47] Harwood CG, Knight CJ. Parenting in youth sport: A position paper on parenting expertise. Psychol Sport Exerc. 2015;16(1):24–35. 10.1016/j.psychsport.2014.03.001.

[48] Siekańska M. Athletes’ perception of parental support and its influence in sports accomplishments– A retrospective study. Hum Mov. 2012;13(4):384–91. 10.2478/v10038-012-0046-x.

